# Construct validity of 2 measures to assess reasons for antipsychotic discontinuation and continuation from patients’ and clinicians’ perspectives in a clinical trial

**DOI:** 10.1186/1471-2288-12-142

**Published:** 2012-09-13

**Authors:** Douglas Faries, Haya Ascher-Svanum, Glenn Phillips, Allen W Nyhuis, Tomoko Sugihara, Virginia Stauffer, Bruce J Kinon

**Affiliations:** 1 Eli Lilly and Company, Lilly Corporate Center, DC 5024, Indianapolis, IN, 46285, USA; 2 Medfocus, LLC, 8600 West Bryn Mawr Avenue, Chicago, IL, 60631, USA

**Keywords:** Schizophrenia, Concurrent validity, Antipsychotic

## Abstract

**Background:**

Little is known about the specific reasons for antipsychotic discontinuation or continuation from patients’ or clinicians’ perspectives. This study aimed to assess the construct validity of 2 new measures of the Reasons for Antipsychotic Discontinuation/Continuation (RAD): RAD-I (a structured interview assessing the patient’s perspective) and RAD-Q (a questionnaire assessing the clinician’s perspective).

**Methods:**

Data were used from a 12-week antipsychotic trial of schizophrenia patients in which the RAD was administered at study entry and at study completion (or discontinuation). Construct validity was assessed through comparisons of RAD responses, clinicians’ responses to a standard patient disposition form identifying reasons for patient’s study discontinuation, and several standard psychiatric measures. Percent agreement quantified the correspondence between patient and clinician scores.

**Results:**

Patients indicating lack of improvement/worsening of positive symptoms as a ‘somewhat’ to ‘primary’ reason for medication discontinuation had statistically significantly less improvement in Positive and Negative Syndrome Scale positive score than patients not reporting these as a reason (concurrent validity). Similar results were observed for the RAD negative symptom, functional, social support, and adherence items, whereas the mood and cognitive items were not significantly associated with change scores on standard psychiatric measures. Responses to the RAD were also weakly associated with variables that theoretically should not be related to them (divergent validity). Level of agreement between the clinician- and patient-rated RAD scores was high (60%-100%).

**Conclusions:**

Initial validation of the RAD suggests that the instruments are valid tools for gathering detailed information regarding reasons for antipsychotic discontinuation and continuation from patients’ and clinicians’ perspectives.

## Background

Treatment discontinuation among patients with schizophrenia is considered a proxy measure of a medication’s effectiveness, reflecting its efficacy, safety, and tolerability from both patients’ and clinicians’ perspectives [1,2]. Both clinical trial and observational research has shown that adherence to antipsychotic treatment is low [1-4]. A recent registry study by Tiihonen et al. [4] reported that only 58.2% of patients in Finland collected a prescription for an antipsychotic during the first 30 days after hospital discharge and only 45.7% continued their initial treatment for 30 days or longer. Although the reasons for medication discontinuation are recognized as being of great clinical importance, prior research has assessed these reasons only at a broad or upper level, focusing on broad categories including lack of medication efficacy, medication intolerability, patient decision, and ‘other’. Furthermore, no information on why patients continue on antipsychotic treatment is typically captured in clinical trials.

Interestingly, although the Clinical Antipsychotic Trials of Intervention Effectiveness (CATIE) schizophrenia study demonstrated that patients discontinued antipsychotic treatment primarily due to ‘patient decision’ (followed by lack of efficacy and intolerability), the main drivers of ‘patients’ decisions’ are unclear. It has been previously noted [5] that because the drivers of ‘patient decision’ are unclear, the true rate of discontinuation due to medication intolerability has likely been underestimated. The idea that ‘patient decision’ might be a proxy for medication intolerability seems incongruent with several studies showing that patients’ perceptions of medication’s benefits and early treatment response are the primary predictors of treatment continuation and discontinuation [6-10]. Importantly, identifying the specific reasons for continuing or discontinuing antipsychotics will allow for a more granular understanding of the specific elements that lead to discontinuation and help clinicians better tailor following treatment to patients’ needs.

In order to address this gap, 2 new measures of the Reasons for Antipsychotic Discontinuation/Continuation (RAD) were developed: The RAD-I (a structured interview assessing the patient’s perspective) and the RAD-Q (a questionnaire assessing the clinician’s perspective) [11,12]. The present study examined the construct validity of the 2 RAD measures using data from a 12-week clinical trial of patients with schizophrenia.

## Methods

### RAD development

Two RAD scales were developed – the RAD-I was designed as a structured interview assessing the patient’s perspective, and the RAD-Q was developed as a questionnaire assessing the clinician’s perspective (see Appendix 1). The items for the preliminary versions of the RAD were selected based on a comprehensive literature review, a patient interview pilot study, and input from an expert working group. Patients with schizophrenia or schizoaffective disorder and their clinicians completed the draft measures and structured cognitive debriefing interviews assessing the measures’ comprehensibility, clarity, and comprehensiveness. The draft measures were further revised following cognitive debriefing interviews with clinicians, patients, and interviewers. A more comprehensive description of the qualitative work undertaken to develop these scales has previously been presented [11,12]. The scales are available from the corresponding author upon request.

Both the RAD-Q and the RAD-I have 2 sections: one to assess reasons for antipsychotic discontinuation (administered only if treatment is being discontinued) and the other to assess reasons for antipsychotic continuation (administered only if treatment is continuing). Briefly, the RAD instruments contain statements related to why a patient or clinician may choose to stop or continue an antipsychotic, such as ‘This medication did not sufficiently improve positive symptoms (e.g., hallucinations, delusions)’; ‘Non-life threatening side effects (Please list up to 5)’; and ‘Financial cost of the medication’. The RAD-I and RAD-Q each include 25 items assessing reasons for medication discontinuation and 21 (RAD-I) or 18 (RAD-Q) items assessing reasons for medication continuation. The RAD-Q is completed by a clinician who responds to whether each item was not a reason, or was a minor, somewhat important, or primary reason for discontinuation/continuation. The RAD-I is completed by interviewing the patient using simple direct questions (e.g., ‘Why did you stop taking the antipsychotic?’). Vague or incomplete responses are queried for specific reasons. The interviewer then maps the patient’s responses onto statements similar to those in the RAD-Q and asks the patient how important each was in the decision to discontinue/continue. The RAD version used in this clinical trial has since undergone some relatively minor changes, particularly in the scoring of the RAD-I. The RAD scales are available upon request from the third author.

### Study design

To examine the validity of the RAD measures, we used data from a randomized, double-blind study (NCT00337662) exploring the relationship between early response to an antipsychotic medication and subsequent improvement in psychopathology. Patients aged 18 years or older with a diagnosis of schizophrenia, schizoaffective disorder, or schizophreniform disorder, according to the Diagnostic and Statistical Manual of Mental Disorders, 4th edition (DSM-IV), were included in the study, provided they met a set of symptom severity criteria at screening.

Patients were treated in an open-label fashion with risperidone (2–6 mg/day) for 2 weeks, at which time early response status was determined in a double-blind manner. Patients who showed early response continued to receive risperidone for the duration of the study. Patients who did not show an early response to risperidone were randomized in a double-blind manner (1:1) to either olanzapine (10–20 mg/day) or risperidone (2–6 mg/day) for 10 weeks. Further details about the study design and core findings are available elsewhere [13].

Written informed consent was obtained from each patient and the study was approved by the Ethics committee from each institution in which it was conducted and the study was done in accordance to the Declaration of Helsinki.

### Study measures

A number of validated standard psychiatric measures were used to assess the validity of the RAD. These measures included the Positive and Negative Syndrome Scale (PANSS) [14,15], Quality of Life Scale (QLS) [16], Modified Schizophrenia Care and Assessment Program Health Questionnaire (SCAP-HQ) [17], and the standard patient disposition form used by clinicians in clinical trials to identify the reason for study discontinuation. The PANSS is one of the most well studied scales for symptom severity in schizophrenia with demonstrated internal consistency, reliability, and validity, multiple factor structure assessments [15,18] and anchoring to other clinical measures [19]. The QLS was designed to assess symptoms of functional deficit in schizophrenia patients, and the initial validation included assessments of construct validity, factor analysis, and inter-rater agreement [16]. The SCAP-HQ was developed to assess outcomes of routine care for patients with schizophrenia, with items drawn from existing measures, and validated in a sample of over 1500 patients assessing the factor structure, internal consistency, convergent validity, responsiveness to change, and test-retest reliability [17].

The RAD-Q and RAD-I were administered at baseline (referring to the medication discontinued just prior to entering the trial) and at Week 12 (referring to the study drug). If a patient discontinued prior to Week 12, the RAD scales were to be completed at study endpoint (referring to the study drug). While some psychiatric measures were administered at every study visit, at a minimum, the above measures were administered at baseline and Week 12 (or study discontinuation), corresponding with RAD administration. Endpoint RAD data was the focus of this current analysis as this was prospectively assessed and allowed for comparison with changes in symptom rating scales captured during the trial.

The standard patient disposition form administered at study endpoint captured the broad or upper-level reason for discontinuation from the physician’s perspective. Options included: adverse event, lack of efficacy, protocol violation, patient decision, physician decision, and parent/caregiver decision. For patients who completed the study (i.e., did not discontinue early), the study staff would select ‘patient completed’ and no additional information was captured.

### Analysis methods

Summary statistics were utilized to describe the demographic and baseline patient characteristics for the study population. To assess the impact of patients without RAD data on the generalizability of our findings, the characteristics of patients with and without RAD data were compared using t- and chi-square tests.

Multiple assessments of concurrent validity utilizing relationships of the RAD and the standard patient disposition measure and relationships between the RAD and symptom severity scales were used to establish construct validity. First, the percentage of times RAD reasons for discontinuation were in agreement with clinicians’ reported reasons for discontinuation using the standard patient disposition form was computed. For instance, patients who discontinued due to an adverse event per standard disposition form were considered to have a matching response on the RAD if the clinician identified (on the RAD-Q) an adverse event as the ‘most important’ reason for discontinuing. In addition, analysis of variance was utilized to assess differences in RAD component scores across these standard disposition form groups.

The validity of each RAD item was assessed by categorizing patients into 2 groups based on their RAD response: patients who responded that the reason was ‘somewhat important’, ‘very important’, or ‘primary’ reason for discontinuation (or continuation) versus patients who responded that the item was ‘not a reason’ for discontinuation (or continuation). These 2 groups were then compared on their differentiation on corresponding symptom scale scores. For example, to assess the RAD item ‘positive symptoms not sufficiently improved’, group differences in change from baseline to discontinuation in PANSS positive symptom scores were assessed. To assess divergent validity, it was hypothesized that effect sizes between the above groups would be stronger for the corresponding scale (e.g., RAD positive items with the PANSS positive subscale) than other measures (e.g., RAD positive items with PANSS negative subscale, PANSS mood subscale, PANSS functional subscale, quality of life, social support, pulse, and weight). Agreement between corresponding questions on the RAD-Q and RAD-I was compared using Pearson’s correlation coefficient, percentage agreement, and the kappa coefficient.

## Results

**Figure 1:**
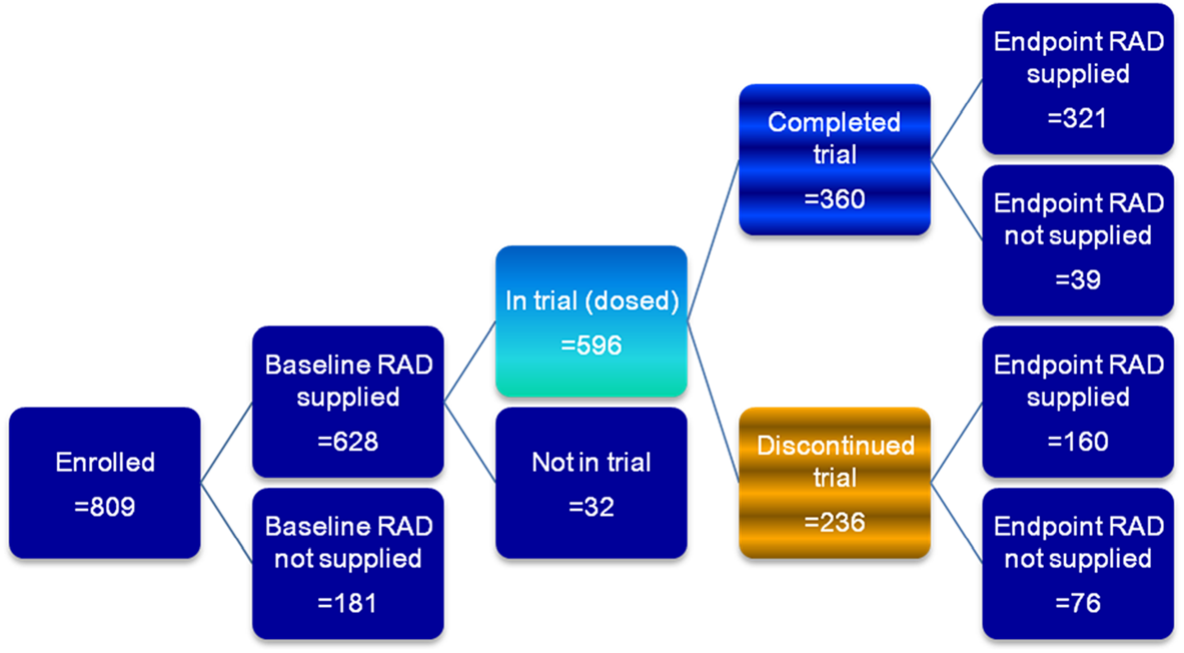
**Overview of patient disposition and RAD data collection.** Abbreviations: RAD = Reasons for Antipsychotic Discontinuation/Continuation.

Figure 1 summarizes the patient disposition and RAD data collection during the study. RAD data were obtained from 160 of the 236 patients who discontinued and from 321 of the 360 patients who completed the trial. Table 1 summarizes the baseline characteristics for patients with baseline and with post-baseline RAD data. Patients had a mean PANSS total score of about 93, and a mean Clinical Global Impressions of severity score of 4.6, indicating being moderately to markedly ill. To assess whether the population of patients with RAD data was representative of the population entering the trial, baseline and demographic data were compared between patients with and without RAD post-baseline data. The populations were similar, with no statistically significant differences.

**Table 1:** Baseline characteristics of patients with baseline and post-baseline data

	**Baseline patient population**	**All patients with post-baseline RAD data**
	**(N = 596)**	**(N = 481)**
Age, mean (SD)	41.8 (11.0)	41.8 (11.0)
Male,%	62.2	61.5
Race,%		
African-American	44.8	44.1
Caucasian	44.1	43.9
Other	11.1	12.1
Hospitalizations in Past Year,%	39.4	38.5
Diagnosis,%		
Schizophrenia	75.5	76.9
Schizoaffective – Bipolar	15.8	14.1
Schizoaffective – Depressive	8.7	8.9
Living Situation,%	57.6	56.5
No/Limited Supervision		
PANSS Total, mean (SD)	92.5 (13.8)	92.3 (13.3)
MADRS Total, mean (SD)	16.2 (9.2)	16.0 (9.3)
CGI-Severity, mean (SD)	4.6 (0.6)	4.6 (0.6)

No statistically significant (p < .05) differences were found between patients with and without a post-baseline RAD measurement.

CGI = Clinical Global Impressions; MADRS = Montgomery-Asberg Depression Rating Scale; N = number of patients; PANSS = Positive and Negative Syndrome Scale; RAD = Reasons for Antipsychotic Discontinuation/Continuation; SD = standard deviation.

The RAD-Q item that was selected as the ‘single most important’ reason for discontinuation more than any other item was ‘insufficient improvement/worsening of positive symptoms’ (24.3%) followed by medication adverse events (22.4%). For approximately 18%, no single reason was selected as the most important reason. The most common reasons for continuing the medication based on the RAD-Q were benefits for positive symptoms (46.7%) and patient perceptions of improvement (e.g., patient believed he/she was now better) (15.4%). For approximately 15% of the patients, no single most important reason was given. Rates of specific reasons for discontinuation and continuation from the patient interview (RAD-I) were similar.

**Figure 2:**
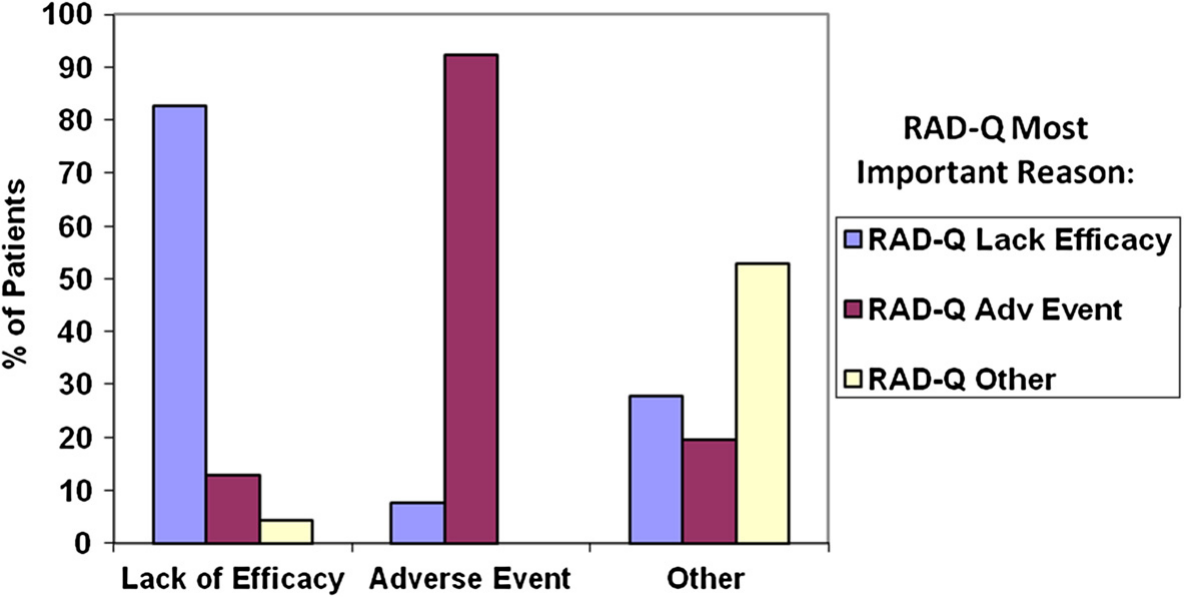
**Assessment of RAD responses (RAD-Q most important reason) stratified by standard form reason for discontinuation post-enrollment.** Abbreviation: RAD-Q = Reasons for Antipsychotic Discontinuation/Continuation questionnaire assessing the clinician’s perspective.

**Figure 3:**
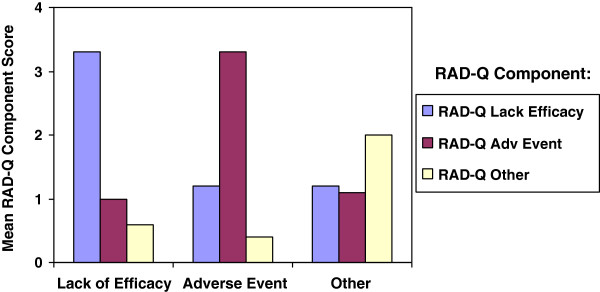
**Assessment of RAD responses (RAD-Q component scores) stratified by standard form reason for discontinuation post-enrollment.** Abbreviation: RAD-Q = Reasons for Antipsychotic Discontinuation/Continuation questionnaire assessing the clinician’s perspective.

Based on the standard patient disposition form, ‘patient decision’ (33.9%, excluding patients who were lost to follow-up) was the most common reason for study discontinuation. Figures 2 and 3 provide an assessment of concurrent validity of the RAD utilizing patient groups based on the standard patient disposition form. Specifically, Figure 2 summarizes the RAD primary reason for discontinuation by reasons stated in the standard patient disposition form. Over three-fourths (82.6%) of patients discontinuing medication due to ‘lack of efficacy’ per the standard trial form had lack of efficacy as the single most important reason for discontinuation on the RAD-Q. Similarly, 92.3% of patients with a standard form reason of ‘adverse event’ identified a RAD-Q tolerability item as the single most important reason. Rates were slightly lower for the RAD-I, though there was still strong concordance. Approximately 50% of those with ‘other’ reasons on the standard form identified adverse events and lack of efficacy reasons on the RAD. ‘Patient decision’ on the standard form reflected a mix of reasons on the RAD: lack of efficacy (42%), intolerability (32%), and other reasons (26%; e.g., non-adherence).

Figure 3 displays the correspondence between RAD discontinuation component scores to the standard patient disposition form discontinuation reason groups. For example, patients with an adverse event reason per the standard form had statistically significantly higher RAD-Q adverse event component scores than patients with ‘lack of efficacy’ or patients with ‘other’ as their standard form reason for discontinuation.

Table 2 summarizes the assessment of concurrent validity for RAD discontinuation items. Patients for whom lack of improvement of positive symptoms was ‘a somewhat important reason’, ‘a very important reason’, or ‘a primary reason’ for medication discontinuation on the RAD-Q and RAD-I had statistically significantly less improvement in PANSS positive score than patients for whom this was not a listed reason. For the RAD-Q, similar results were observed for negative symptoms (relative to PANSS negative scores), mood (relative to PANSS Anxiety/Depression), functioning (relative to QLS total), social support (relative to QLS interpersonal relations), and adherence (relative to SCAP adherence). For the RAD-I, similar results were observed for cognition, functioning, and social support, though no significant differences were found for mood.

**Table 2:** Change in symptom domains per standard validated measures by RAD reasons for study drug discontinuation

			**Groups based on RAD-Q (Clinicians)**			**Groups based on RAD-I (Patients)**		
**RAD domain**	**Item**	**Comparator**	**N**	**Mean change (SE)**	**p-value**	**N**	**Mean change (SE)**	**p-value**
Positive Symptoms	Not Sufficiently Improved	PANSS Positive						
Yes			56	−3.2 (0.8)		43	−3.5 (0.8)	
No			103	−6.8 (0.5)	.001	117	−6.3 (0.5)	.003
Positive Symptoms	Worsened	PANSS Positive						
Yes			13	−1.8 (2.2)		16	−2.1 (1.6)	
No			149	−5.9 (0.4)	.089	144	−6.1 (0.4)	.004
Negative Symptoms	Not Sufficiently Improved	PANSS Negative						
Yes			30	−0.8 (0.8)		23	−2.5 (1.0)	
No			124	−3.4 (0.5)	.012	132	−2.9 (0.4)	.738
Negative Symptoms	Worsened	PANSS Negative						
Yes			6	−0.8 (1.3)		8	2.5 (1.1)	
No			155	−2.9 (0.4)	.330	147	−2.9 (0.4)	.003
Mood Symptoms	Not Sufficiently Improved	PANSS Anxiety/Depression						
Yes			24	−0.7 (0.8)		18	−2.2 (1.1)	
No			138	−2.6 (0.3)	.031	130	−2.4 (0.4)	.866
Mood Symptoms	Worsened	PANSS Anxiety/Depression						
Yes			13	−0.4 (1.8)		13	−2.2 (1.1)	
No			149	−2.5 (0.3)	.273	146	−2.6 (0.3)	.743
Cognition	Not Sufficiently Improved	PANSS Cognition						
Yes			16	−2.7 (1.3)		22	−0.6 (1.0)	
No			142	−3.2 (0.4)	.712	136	−3.4 (0.4)	.014
Cognition	Worsened	PANSS Cognition						
Yes			7	−2.1 (1.5)		14	0.8 (1.5)	
No			158	−3.1 (0.4)	.607	145	−3.3 (0.4)	.004
Functioning	Not Sufficiently Improved	QLS Total Score						
Yes			21	−7.7 (4.8)		18	−7.6 (5.1)	
No			135	4.6 (1.5)	.005	136	3.8 (1.5)	.015
Functioning	Worsened	QLS Total Score						
Yes			9	−15.2 (7.4)		12	−14.6 (6.2)	
No			152	3.7 (1.4)	.003	145	3.6 (1.4)	.001
Social Support	Friend/Family Did Not Support	QLS Interpersonal Relations						
Yes			6	−9.0 (3.5)		10	−4.3 (2.9)	
No			154	1.8 (0.7)	.003	158	2.3 (0.7)	.021
General Symptoms	Subject Believed Symptoms Worse	PANSS Total Score						
Yes			21	0.6 (4.4)				
No			142	−17.3 (1.4)	.001			
Adherence	Subject Not Adhering to Medication	SCAP Adherence Item						
Yes			11	2.0 (0.3)				
No			133	1.4 (0.1)	.023			

Yes = “Somewhat” to “Primary Reason” was chosen; No = “Not a Reason” was chosen; N = number of patients; PANSS = Positive and Negative Syndrome Scale; QLS = Heinrich Carpenter Quality of Life Scale; RAD = Reasons for Antipsychotic Discontinuation/Continuation; SCAP = Schizophrenia Care and Assessment Program; SE = standard error.

Table 3 summarizes the assessment of concurrent validity for RAD continuation items. Patients for whom ‘benefits for positive symptoms’ on the RAD-Q or RAD-I were ‘a somewhat important reason’, ‘a very important reason’, or ‘a primary reason’ for continuing the medication had statistically significantly greater improvement in PANSS positive symptom scores relative to patients who indicated positive symptoms were ‘not a reason’ for continuing. Similar results were observed for the RAD negative symptom question, mood, cognition, and functioning, though no significant differences were observed for social support.

**Table 3:** Change in symptom domains per standard validated measures by RAD reasons for study drug continuation

			**Groups based on RAD-Q**			**Groups based on RAD-I**		
**RAD domain**	**Item**	**Comparator**	**N**	**Mean change (SE)**	**p-value**	**N**	**Mean change (SE)**	**p-value**
Positive Symptoms	Benefits	PANSS Positive						
Yes			215	−10.4 (0.4)		206	−10.7 (0.4)	
No			20	−5.2 (1.5)	.001	30	−5.6 (0.8)	.001
Negative Symptoms	Benefits	PANSS Negative						
Yes			111	−7.8 (0.5)		110	−7.6 (0.5)	
No			90	−2.9 (0.5)	.001	112	−3.5 (0.5)	.001
Mood Items	Benefits	PANSS Anxiety/Depression						
Yes			113	−5.7 (0.4)		139	−5.5 (0.3)	
No			97	−3.4 (0.4)	.001	83	−3.3 (0.4)	.001
Cognition Items	Benefits	PANSS Cognition						
Yes			108	−7.7 (0.5)		131	−7.1 (0.5)	
No			102	−4.0 (0.4)	.001	91	−4.1 (0.4)	.001
Functioning Items	Benefits	QLS Total						
Yes			137	25.1 (2.0)		141	23.4 (2.0)	
No			81	7.3 (2.0)	.001	88	8.5 (2.0)	.001
Social Support Items	Family/Friends Support	QLS Interpersonal Relations						
Yes			43	7.0 (1.4)		45	7.6 (1.3)	
No			181	8.1 (0.8)	.512	186	7.7 (0.8)	.975

Yes = “Somewhat” to “Primary Reason” was chosen; No = “Not a Reason” was chosen; N = number of patients; PANSS = Positive and Negative Syndrome Scale; QLS = Heinrich Carpenter Quality of Life Scale; RAD = Reasons for Antipsychotic Discontinuation/Continuation; SE = standard error.

To assess divergent validity, differences between the analysis groups in Tables 2 and 3 were assessed using scales hypothesized to have a lesser relationship with the RAD item. Patients for whom the RAD-Q indicated positive symptoms were a ‘somewhat’ to ‘primary’ reason for discontinuation (compared to those indicating positive symptoms were not a reason) had a stronger relationship with PANSS positive symptoms (effect size = 0.66) than with all other PANSS subscales, the QLS, and with physiologic outcomes (i.e., weight, pulse; absolute effect size range of 0.14 to 0.59). Similarly, for the RAD-I assessment of positive symptoms, the group effect size was 0.54 for the PANSS positive subscale and ranged from 0.08 to 0.44 for other measures.

Agreement between the corresponding items on the clinician (RAD-Q) and patient-interview (RAD-I) based scores was high (range 60% to 100%) for all items (see Table 4). Agreement was slightly higher for discontinuation items relative to continuation items and higher for items covering issues other than efficacy and safety, such as cost, insurance, and social support. Correlations were also high (r > .5) except for the ‘unable to connect with members of the treatment team’ item. Chance-corrected agreement (Kappa coefficients) ranged from 0.42 to 1.00 (see Table 4). All coefficients are at least moderately high as defined by Landis and Koch [20]. 

**Table 4:** Agreement between corresponding items on the RAD-Q and RAD-I

	**Discontinuation items**			**Continuation items**		
**RAD item**	**% Agreement**	**Pearsons’s correlation (r)**	**Kappa coefficients**	**% Agreement**	**Pearsons’s correlation (r)**	**Kappa coefficients**
Positive	86.3	0.75	0.61	67.7	0.71	0.53
Negative	87.7	0.64	0.54	60.2	0.68	0.46
Mood	86.2	0.66	0.52	60.3	0.68	0.47
Cognition	87.2	0.61	0.54	60.6	0.74	0.47
Functioning	89.4	0.70	0.55	67.2	0.82	0.56
Adverse Event	79.8	0.94	0.72	63.4	0.67	0.49
Finances	97.7	0.91	0.82	87.9	0.67	0.59
Insurance	99.4	0.91	0.91	88.8	0.75	0.62
Health System	98.3	0.91	0.62	80.7	0.63	0.51
Transportation	100.0	1.00	1.00	85.0	0.67	0.52
Social Support	96.7	0.82	0.66	79.0	0.71	0.52
Believed Better	95.5	0.80	0.45	61.9	0.65	0.49
Unable to Connect	98.3	0.30	0.57	70.7	0.77	0.55
Influence Another	93.7	0.62	0.62	87.0	0.57	0.50
Try New Medication	97.7	0.64	0.49	70.7	0.74	0.55

## Discussion

This study provided evidence supporting the construct validity of the first instruments developed to assess the specific reasons for antipsychotic discontinuation or continuation from patients’ and clinicians’ perspectives. This validation study, using data from a 12-week clinical trial, supports the RAD as a valid tool in psychiatric research and suggests it may also be of utility in clinical practice. Strong agreement was observed between the reasons clinicians identified on the RAD and the reasons they identified on the standard patient disposition form. Significant differences in the RAD component scores were found between patient groups based on the standard disposition form. In addition, patient groups based on the RAD differentiated as hypothesized on validated standard psychiatric measures. Furthermore, agreement between the clinician- and patient-rated RAD scores was high, suggesting that patients’ and clinicians’ perspectives on antipsychotic treatment discontinuation and continuation are reasonably similar. However, the design of the trial did not require independent scorers for the 2 scales and thus conclusions regarding the agreement are somewhat limited.

The results of these analyses are similar to those of Weiden et al. [21] who developed the Rating of Medication Influences (ROMI), a standardized measure for assessing attitudinal and behavioral factors influencing patient compliance with antipsychotic medications. Analyses to assess the psychometric properties of the ROMI demonstrated that the ROMI is a reliable and valid instrument to assess the subjective reasons for medication compliance and noncompliance.

Lack of efficacy (specifically relating to positive symptoms), rather than adverse events, was found to be the main driver of treatment discontinuation in this study from both the clinicians’ and patients’ perspectives. Current findings are in agreement with previous research [6-10] which suggests that lack of efficacy is the main driver of medication discontinuation in patients with schizophrenia. Study results also provide, for the first time, important information on the specific reasons that underlie a large but ambiguous discontinuation category: ‘patient decision.’ Such patients were identified on the RAD as having discontinued due to a mix of reasons, led by lack of efficacy (42%) and followed by medication intolerability (32%).

Results also suggest that improvement of positive symptoms is the most common single reason for continuing antipsychotic medication (from both clinician and patient perspectives). Although little research is available on the reasons for continuing medication, this is in agreement with Liu-Seifert et al. [8,9] who found that patients’ perceptions of a beneficial effect of antipsychotic treatment, assessed using the ROMI, may be a significant factor in treatment persistence and satisfactory treatment outcome.

Understanding the specific reasons for medication discontinuation is of vital importance in usual care, where switching of treatment regimen is prevalent. While there is research supporting the benefits of switching when warranted [22-24], others have challenged this notion [25]. The CATIE study has given rise to multiple efforts to provide guidance on medication switching [26-29]. However, these and other research [5] suggest that prior medication and the reason for medication switching are important factors in determining the optimal next steps in treatment for the individual patient. Thus, a tool such as the RAD, which can provide greater specificity regarding reasons for medication change (or lack of change) could be valuable in both furthering research and improving clinical decision-making.

The current study has several limitations. First, data for this study were derived from a clinical trial, and thus may not be generalizable to patients with schizophrenia treated in clinical practice. To address this gap, another study, assessing the validity of the RAD in usual care settings, is in progress. Second, this trial enrolled acutely exacerbated psychotic patients with schizophrenia who had a mean PANSS score of about 93 at baseline. As a result, patients and clinicians may have placed a premium on the amelioration of psychotic symptoms and may have focused on positive symptoms. However, a recent study that included patients who were less severely ill demonstrated that continuation and discontinuation appear driven by the medication’s efficacy for improving positive symptoms [30]. Third, the RAD has not been evaluated outside the United States and until additional research is conducted, its validity in other geographic regions is uncertain. Fourth, this trial utilized preliminary versions of the RAD. While ongoing development of the RAD resulted in only limited revisions, confirmation of these results is needed. Fourth, a relatively small number of patients discontinued from the clinical trial and some of these did not provide RAD data, which limited the analysis on reasons for medication discontinuation. Finally, the RAD is the first measure designed to assess specific reasons for medication discontinuation or continuation from both patients’ and clinicians’ perspectives and, thus, our lack of ability to compare the instruments with an established and validated ‘gold standard’. The RAD is applicable for assessing discontinuation/continuation of antipsychotics for the treatment of schizophrenia and was not designed for other disease states and medications. However, the model used to develop and validate the RAD, beginning with patient interviews and expert working groups, could be of significant value in other areas where medication adherence is critical.

## Conclusions

This research provided support for the construct validity of the RAD in a clinical trial population. Additional research is needed to clarify whether the RAD, when not used in the study of schizophrenia patients with acute exacerbations, would provide similar results. Despite current limitations, it appears these are valid instruments to assess an important driver of treatment outcomes, namely the patients and their physicians’ wish to continue or discontinue a given antipsychotic treatment regimen.

## Appendix 1

The Reasons for Antipsychotic Discontinuation/Continuation (RAD) scales were developed to assess the specific reasons for antipsychotic discontinuation or continuation. The RAD-I is a structured interview assessing the patient's perspective and the RAD-Q a questionnaire assessing the clinician's perspective. Table 5 provides the specific questions and response options for both the discontinuation and continuation portions of the RAD-Q.Item ScoringA: Yes/No; If Yes, then choose one of the 4 options:1) A minor reason; 2) a somewhat important reason; 3) a very important reason; 4) a primary reasonB: Yes/No; If Yes, then ‘Which item?’

**Table 5:** Reasons for Antipsychotic Discontinuation/Continuation questionnaire assessing the clinician’s perspective (RAD-Q)

**Item**	**Scoring**	**Item**	**Scoring**
**RAD-Q Discontinuation**		**RAD-Q Continuation**	
1. This medication did not sufficiently improve positive symptoms (e.g., hallucinations, delusions).	A	30. The medication has no serious safety issues that are dangerous and potentially life-threatening for this patient (e.g., seizures, heart arrhythmia, agranulocytosis).	A
2. This medication made positive symptoms worse.	A	31. Benefits for positive symptoms (e.g., hallucinations, delusions).	A
3. This medication did not sufficiently improve negative symptoms (e.g., flat affect, lack of motivation).	A	32. Benefits for negative symptoms (e.g., flat affect, lack of motivation).	A
4. This medication made negative symptoms worse.	A	33. Benefits for the patient’s mood (e.g., depression).	A
5. This medication did not sufficiently improve the patient’s mood (e.g., depression).	A	34. Benefits for cognition (e.g., planning, attention, memory).	A
6. The medication made the patient’s mood worse.	A	35. Benefits for functional status (e.g., self-care activities of daily living, or work).	A
7. This medication did not sufficiently improve cognition (e.g., planning, attention, memory).	A	36. Financial cost of medication.	A
8. This medication made cognition worse.	A	37. The patient’s insurance adequately covers this medication.	A
9. This medication did not sufficiently improve functional status (e.g., the patient’s ability to work or live independently).	A	38. The patient is willing/able to negotiate the health-care system to obtain this drug (e.g., getting prescriptions filled, scheduling/attending appointments).	A
10. This medication made functional status worse.	A	39. There are no problems with transportation (e.g., getting to the pharmacy to refill medication).	A
11. A medication-related serious safety issue that was dangerous and potentially life-threatening (e.g., seizures, heart arrhythmia, agranulocytosis).	A	40. Social support (e.g., friends or family support the patient in taking this medication).	A
12-16. Please list up to 5 non-life threatening side effects experienced by the patient that were reasons for discontinuing this medication.	A	41. Patient perceptions of improvement (e.g., the patient believed he/she was now “better” and wants to continue taking the medication).	A
17. Financial cost of the medication.	A	42. The patient has formed a therapeutic alliance or connection with members of the treatment team.	A
18. The patient’s insurance did not adequately cover this medication.	A	43. Another person told this patient to continue taking the medication (if so, what is the relationship of this person to the patient?).	A
19. Difficulty negotiating the health-care system (e.g., getting prescriptions filled, scheduling/attending appointments).	A	44. The patient has already tried other antipsychotics that have not been as effective and/or tolerable.	A
20. Problems with transportation (e.g., getting to the pharmacy to refill medication).	A	45-46. Other: Specify.	
21. Social support (e.g., friends or family did not support patient in taking this medication).	A	47-51. Please list up to 5 side effects the patient experiences from this antipsychotic.	A
22. Patient believes he/she no longer needed the medication because he/she was now “better.”	A	Of all the reasons listed in items 30–51, is there one that you consider to be the most important reason for continuing this medication?	B
23. The patient believed the medication was causing symptoms to become worse.	A		
24. The patient was unable to connect with members of the treatment team.	A		
25. Another person told this patient to stop the medication (if so, what is the relationship of the person to the patient?).	A		
26. The patient wished to try an antipsychotic new to the market.	A		
27. Potential interactions with another drug prescribed for this patient?	A		
28. The patient was not adhering to the medication regimen.	A		
29. The patient developed a new medical condition and this antipsychotic may have exacerbated the condition.	A		
Of all the reasons listed in items 1–29, is there one that you consider to be the most important reason for discontinuing this medication?	B		

## Abbreviations

CATIE: Clinical Antipsychotic Trials of Intervention Effectiveness; DSM-IV: Diagnostic and Statistical Manual of Mental Disorders 4^th^ edition; PANSS: Positive and negative syndrome scale; QLS: Quality of Life Scale; RAD: Reasons for Antipsychotic Discontinuation/Continuation; RAD-I: Reasons for Antipsychotic Discontinuation/Continuation interview assessing the patient’s perspective; RAD-Q: Reasons for Antipsychotic Discontinuation/Continuation questionnaire assessing the clinician’s perspective; ROMI: Rating of Medication Influences; SCAP-HQ: Schizophrenia Care and Assessment Program Health Questionnaire.

## Competing interests

Doug Faries, Haya Ascher-Svanum, Glenn Phillips, Allen Nyhuis, Virginia Stauffer, and Bruce Kinon were all full-time employees and minor shareholders of Lilly at the time this research was conducted. Tomoko Sugihara was a contractor for Lilly and employed by MedFocus.

## Authors’ contributions

Authors DF, HA-S, GP, VS, and BJK contributed to the conception and design of the study; DF, AWN, and TS contributed to the analysis and interpretation of data, DF and HA-S drafted the manuscript, and all authors critically revised the manuscript for intellectual content, and have approved the final version of the manuscript.

## Pre-publication history

The pre-publication history for this paper can be accessed here:

http://www.biomedcentral.com/1471-2288/12/142/prepub
